# Ilixadencel, a Cell-based Immune Primer, plus Sunitinib Versus Sunitinib Alone in Metastatic Renal Cell Carcinoma: A Randomized Phase 2 Study

**DOI:** 10.1016/j.euros.2022.03.012

**Published:** 2022-04-26

**Authors:** Magnus Lindskog, Anna Laurell, Anders Kjellman, Bohuslav Melichar, Pablo Maroto Rey, Henryk Zieliński, Felipe Villacampa, Pierre Bigot, Bajory Zoltan, Omi Parikh, David Vazquez Alba, Åsa Jellvert, Tibor Flaskó, Enrique Gallardo, Maria José Ribal Caparrós, Gunta Purkalne, Peter Suenaert, Alex Karlsson-Parra, Börje Ljungberg

**Affiliations:** a Department of Immunology, Genetics and Pathology, Uppsala University Hospital, Uppsala University, Uppsala, Sweden; b Department of Oncology, Akademiska University Hospital, Uppsala, Sweden; c Department of Urology, CLINTEC, Karolinska University Hospital, Karolinska Institutet, Stockholm, Sweden; d Department of Urology and Urological Oncology, Wojewodzki Szpital Specjalistyczny im. Stefana Kardynała Wyszyńskiego, Lublin, Poland; e Hospital de la Santa Creu i Sant Pau, Barcelona, Spain; f Clinical Urology, Military Institute of Medicine, Warsaw, Poland; g Urology Service, Hospital Unversitario 12 Octubre, Madrid, Spain; h Department of Urology, Centre Hospitalier Universitaire d'Angers, Angers Cedex, France; i Szent-Györgyi Albert Klinikai Központ, Szegedi Tudomanyegyetem Altalanos Ovostudomanyi Kar Urologiai Klinika, Szeged, Hungary; j Rosemere Cancer Centre, Royal Preston Hospital, Preston, UK; k Servicio de Urología, Hospital Universitario Puerta de Hierro Majadahonda, Madrid, Spain; l Department of Oncology, Institute of Clinical Sciences, Sahlgrenska Academy, University of Gothenburg, Gothenburg, Sweden; m Department of Urology, Medical School, University of Debrecen, Debrecen, Hungary; n Oncology Department, Parc Taulí Hospital Universitari, Institut d’Investigació i Innovació Parc Taulí I3PT, Universitat Autònoma de Barcelona, Sabadell, Spain; o Department of Urology, Hospital Clínic Barcelona, Barcelona, Spain; p Oncology Clinic, Pauls Stradins Clinical University Hospital, Rīga, Latvia; q Immunicum AB, Stockholm, Sweden; r Department of Immunology, Genetics and Pathology, Section of Clinical Immunology, Uppsala University, Uppsala, Sweden; s Department of Surgical and Perioperative Sciences, Urology and Andrology, Umeå University, Umeå, Sweden

**Keywords:** Allogeneic dendritic cells, Ilixadencel, Intratumoral administration, Metastatic renal cell carcinoma, Off the shelf, Phase 2 trial, Randomized, Sunitinib

## Abstract

**Background:**

The prognosis of patients with synchronous metastatic renal cell carcinoma (mRCC) is poor. Whereas single-agent tyrosine kinase inhibition (TKI) is clearly insufficient, the effects can be enhanced by combinations with immune checkpoint inhibitors. Innovative treatment options combining TKI and other immune-stimulating agents could prove beneficial.

**Objective:**

To evaluate the clinical effects on metastatic disease when two doses of allogeneic monocyte-derived dendritic cells (ilixadencel) are administrated intratumorally followed by nephrectomy and treatment with sunitinib compared with nephrectomy and sunitinib monotherapy, in patients with synchronous mRCC.

**Design, setting, and participants:**

A randomized (2:1) phase 2 multicenter trial enrolled 88 patients with newly diagnosed mRCC to treatment with the combination ilixadencel/sunitinib (ILIXA/SUN; 58 patients) or sunitinib alone (SUN; 30 patients).

**Outcome measurements and statistical analysis:**

The primary endpoints were 18-mo survival rate and overall survival (OS). A secondary endpoint was objective response rate (ORR) assessed up to 18 mo after enrollment. Statistic evaluations included Kaplan-Meier estimates, log-rank tests, Cox regression, and stratified Cochran-Mantel-Haenszel tests.

**Results and limitations:**

The median OS was 35.6 mo in the ILIXA/SUN arm versus 25.3 mo in the SUN arm (hazard ratio 0.73, 95% confidence interval 0.42–1.27; *p* = 0.25), while the 18-mo OS rates were 63% and 66% in the ILIXA/SUN and SUN arms, respectively. The confirmed ORR in the ILIXA/SUN arm were 42.2% (19/45), including three patients with complete response, versus 24.0% (six/25) in the SUN arm (*p* = 0.13) without complete responses. The study was not adequately powered to detect modest differences in survival.

**Conclusions:**

The study failed to meet its primary endpoints. However, ilixadencel in combination with sunitinib was associated with a numerically higher, nonsignificant, confirmed response rate, including complete responses, compared with sunitinib monotherapy.

**Patient summary:**

We studied the effects of intratumoral vaccination with ilixadencel followed by sunitinib versus sunitinib only in a randomized phase 2 study. The combination treatment showed numerically higher numbers of confirmed responses, suggesting an immunologic effect.

## Take Home Message

Intratumoral injection of ilixadencel, consisting of proinflammatory allogeneic dendritic cells, before nephrectomy, followed by sunitinib treatment after nephrectomy showed a trend for improved tumor response rate and overall survival, compared with sunitinib monotherapy.

## 1 Introduction

Metastatic renal cell carcinoma (mRCC) remains a clinical challenge despite recent developments in oncologic treatment option. In particular, improved treatment is urgently needed for the 17–20% individuals diagnosed with synchronous metastases and classified as poor- or intermediate-risk patients according to the International Metastatic Renal Cell Carcinoma Database Consortium (IMDC) [1], [2].

Tyrosine kinase inhibitors (TKIs) targeting vascular endothelial growth factor (VEGF) and related tyrosine kinases have been the cornerstone of medical treatment for mRCC until recently [3], [4]. However, most patients treated with a TKI agent as monotherapy ultimately develop resistance. The era of immune checkpoint inhibitors (ICIs) has recently opened new treatment perspectives, and combination treatments with ICIs with or without a TKI are now recommended as first-line treatment [5], [6], [7], [8], [9], [10].

Ilixadencel is a cell-based, off-the-shelf investigational drug product based on allogeneic monocyte-derived inflammatory cells aimed to prime an adaptive anticancer immune response by recruiting and activating endogenous cross-presenting dendritic cells (DCs) when injected intratumorally [11], [12], [13]. Preclinical in vitro studies with ilixadencel have shown that ilixadencel produces factors that induce phenotypic maturation of allogeneic bystander DCs and enhances their ability to cross-present cell-associated antigens to CD8^+^ T cells [12], [13]. Moreover, local subcutaneous injection of allogeneic mouse-ilixadencel in mice has been shown to induce migration of endogenous DCs to draining lymph nodes and systemic activation of antigen-specific CD8^+^ T cells [11]. In line with these preclinical data, the majority of evaluable patients (11/15) with hepatocellular carcinoma (HCC) treated with intratumoral injections of ilixadencel [14] showed an increased frequency of interferon-gamma–producing CD8^+^ T cells specific for the HCC-associated tumor antigens hTERT and/or AFP. The initial phase 1 data in 12 patients with newly diagnosed synchronous mRCC suggested that ilixadencel treatment before nephrectomy followed by standard sunitinib treatment after nephrectomy was feasible [15]. Based on these results, we designed a randomized, phase 2, open-label, multicenter trial comparing intratumoral ilixadencel before nephrectomy followed by sunitinib with standard of care sunitinib monotherapy after nephrectomy, in patients with synchronous mRCC in the first-line setting.

## 2 Patients and methods

### 2.1 Study design

Patients were randomized in a 2:1 ratio to ilixadencel (two intratumoral doses of ilixadencel in the primary tumor before nephrectomy) followed by sunitinib after nephrectomy (ILIXA/SUN group) or after nephrectomy sunitinib monotherapy (SUN group). Sunitinib treatment was initiated 5–8 wk after nephrectomy. Patients were followed on for 18 mo from randomization unless early discontinuation due to disease progression, intolerance to therapy, death, or withdrawal of consent. After study survival follow-up, with no other data collection, is ongoing and extends to 5 yr after study or until the date of death, whichever occurs first. The minimum follow-up time was 36.2 mo for patients still alive. The last follow-up was performed in January 2021. The trial was conducted at 26 sites in the Czech Republic, France, Hungary, Latvia, Poland, Spain, Sweden, the UK, and the USA. The trial was approved by the institutional review board of each participating center. This study is registered with the European Clinical Trials (EudraCT) database (2014-004510-28) and the ClinicalTrials.gov (NCT02432846).

### 2.2 Patients

Eligible patients were 18 yr of age or older with newly diagnosed synchronous mRCC with at least one computed tomography (CT)-verified metastasis (≥10 mm in the longest diameter) for whom complete metastasectomy was not considered feasible. All patients had Eastern Cooperative Oncology Group (ECOG) performance status (PS) of 0–2 and adequate end-organ and bone marrow function, and were without any significant comorbidities. Patients with known brain metastases following metastasis-directed local treatment were eligible provided that there were no indications of either clinical or radiological progression before the initiation of the study.

The patients provided written informed consent.

### 2.3 Randomization

Patients were stratified according to the IMDC criteria (high vs intermediate risk) and randomized in a 2:1 ratio to receive either ilixadencel plus sunitinib or sunitinib alone. Treatment allocation was not masked, and patients received information about study design and the allocated treatment.

### 2.4 Procedures

Cryo-preserved ilixadencel [12], provided by Immunicum AB (Stockholm, Sweden), was injected intratumorally (ultrasound or CT guided) into the primary kidney tumor twice, 2 wk apart, with 10 million viable, HLA-DR–expressing cells per dose, followed by nephrectomy at least 3 d after the second vaccine dose and within 56 d from randomization. Sunitinib was administered orally using the standard regimen once per day at a dose of 50 mg for 4 wk, followed by a 2-wk break. Adverse events (AEs) were managed with treatment interruptions or dose reductions. Sunitinib dose could be reduced to 37.5 and 25 mg. CT scans for central assessment of tumor response were performed at screening, sunitinib start (ie, 5–8 wk after nephrectomy [baseline]), each sunitinib follow-up visit (6, 12, 24, 36, 48, and 60 wk), and end-of-study visit (18 mo; Supplementary Fig. 1). Response assessment was based on an independent blinded central review and response evaluation criteria in solid tumors version 1.1 (RECIST 1.1) [16]. Treatment was continued until disease progression, intolerance to therapy, or withdrawal of consent.

### 2.5 Outcomes

The coprimary endpoints were 18-mo survival rate and overall survival (OS), both for intention-to-treat and separately by IMDC risk groups. OS was defined as the time from the date of randomization to the time of death from any cause or to the last follow-up for alive patients. The key secondary endpoints were objective response rate (ORR; independent blinded central review), confirmed ORR (amended post hoc analysis based on independent blinded central review data), and progression-free survival (PFS), defined as the time from sunitinib start to the time of progressive disease according to RECIST 1.1 [16] or death following sunitinib initiation from any cause, whichever occurred first. Safety assessment included all randomized patients. Safety data were monitored by an independent data and safety monitoring committee.

### 2.6 Statistical analysis

As this study was exploratory, sample size was not based on power calculation for confirming efficacy. Statistical hypothesis testing was used in the context of exploratory analysis for the primary and secondary endpoints. Time to event endpoints (OS, PFS, and time to response) were evaluated with Kaplan-Meier estimates, and differences analyzed using log-rank test and Cox regressions were applied to estimate hazard ratios (HRs) with corresponding 95% confidence intervals (CIs). Between-group differences in the percentage of patients with an objective response were evaluated with a stratified Cochran-Mantel-Haenszel test. A *p* value of ≤0.05 was considered significant.

## 3 Results

### 3.1 Patient characteristics

**Fig. 1:**
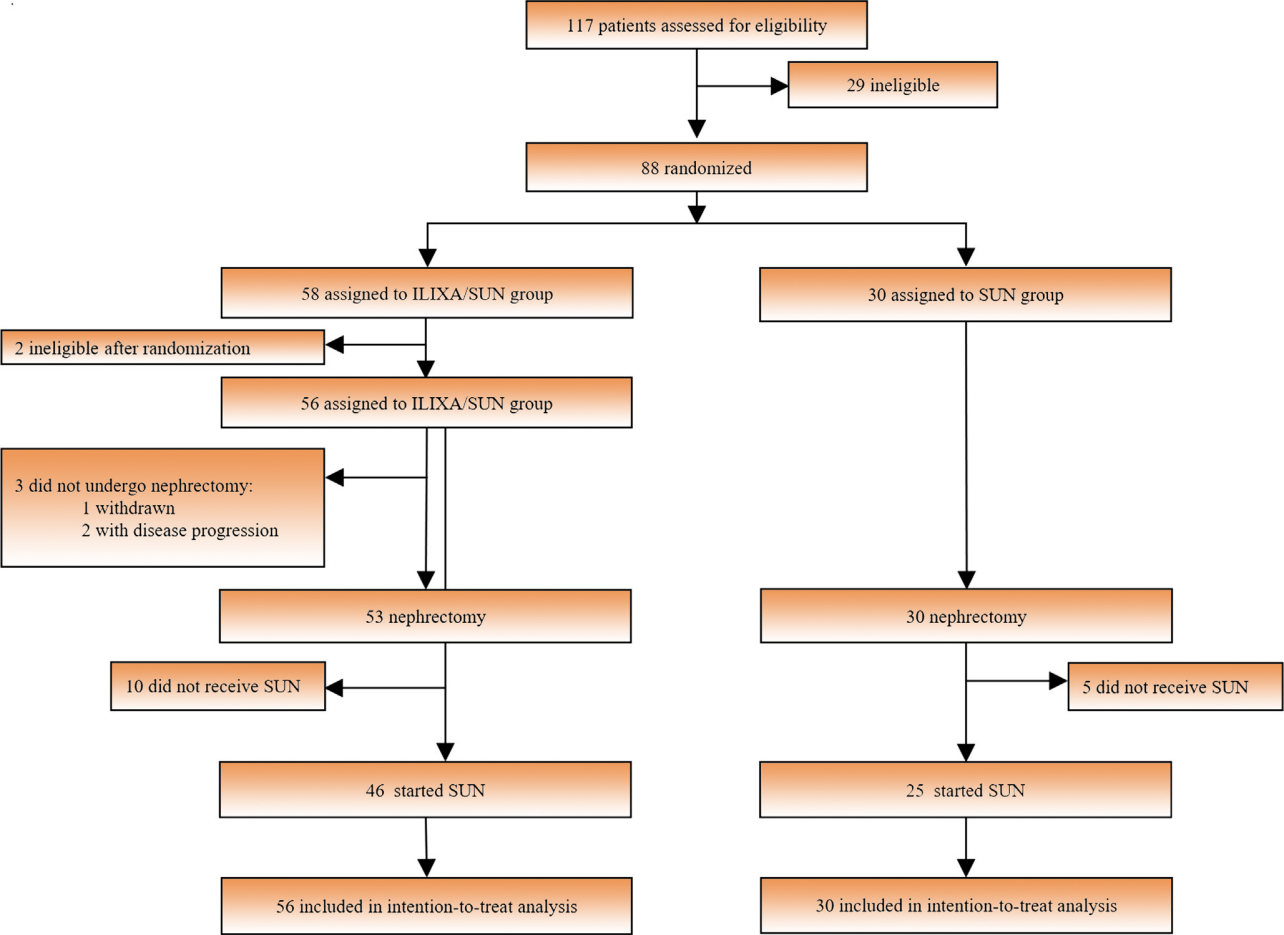
Overall trial profile. During enrollment, 111 patients were assessed for eligibility; 29 patients were screening failures and 88 were randomized. Patients were stratified according to the Heng criteria (high and intermediate risk). Fifty-eight patients (17 high-risk and 41 intermediate-risk patients) were allocated to receive ilixadencel (ILIXA) before nephrectomy and sunitinib (SUN) after nephrectomy (defined as the ILIXA/SUN group), whereas 30 patients (eight high-risk and 22 intermediate-risk patients) were allocated to sunitinib (SUN) alone after nephrectomy (defined as the SUN group).

From April 2014 to January 2017, 88 patients (58 ILIXA/SUN and 30 SUN) were assigned (safety analysis population). Two of the patients initially assigned to the ILIXA/SUN arm were excluded from the full analysis due to withdrawal prior to the first dose of ilixadencel. The intention-to-treat population consisted of 56 patients (16 poor-risk and 40 intermediate-risk patients) for the ILIXA/SUN group and 30 patients (eight poor-risk and 22 intermediate-risk patients) in the SUN group. The overall trial profile is depicted in Fig. 1.

Overall, the treatment groups were balanced with respect to baseline demographic and disease characteristics (Table 1).

**Table 1:** Baseline demographics and clinical characteristics

	ILIXA/SUN (*n* = 56)	SUN (*n* = 30)
Gender, *n* (%)		
Male	43 (76.8)	21 (70)
Female	13 (23.2)	9 (30)
Age (yr)		
Median	63	64
Range	41–76	49–86
Ethnic origin, *n* (%)		
Caucasian	55 (98.2)	28 (93.3)
Unknown	1 (1.8)	2 (6.7)
ECOG performance status (screening), *n* (%)		
0	33 (58.9)	17 (56.6)
1	23 (41.1)	12 (40)
2	0 (0)	1 (3.3)
Time from diagnosis to screening (mo)	0.6	0.8
Tumor type, *n* (%)		
RCC histology	51 (91.1)	27 (90.0)
RCC with clear cell component	49 (87.5)	25 (83.3)
RCC without clear cell component	1 (1.8)^a^	0 (0)
Non-RCC histology	1 (1.8)^b^	1 (3.3)^c^
Clinical and radiological RCC/no nephrectomy due to rapid progression/no histology	4 (7.1)	2 (6.7)
IMDC risk category, *n* (%)		
Intermediate	40 (71.4)	22 (73.3)
Poor	16 (28.6)	8 (26.6)

ECOG = Eastern Cooperative Oncology Group; ILIXA = ilixadencel; IMDC = International Metastatic Renal Cell Carcinoma Database Consortium; RCC = renal cell carcinoma; SUN = sunitinib.

a Chromophobe RCC.

b Sarcoma.

c Urothelial cancer.

In the ILIXA/SUN group (*n* = 56), ten patients never received sunitinib and one patient did not undergo follow-up CT. In the SUN group, five patients never received sunitinib. Death within 2 mo after surgery was the most frequent reason for not starting sunitinib (five patients in the ILIXA/SUN arm and three in the SUN arm). Additional reasons included disease progression (*n* = 3), withdrawal (*n* = 1), different diagnosis at laparotomy (*n* = 1), start of another treatment after nephrectomy (*n* = 1), and withdrawal of consent (*n* = 1). The safety population included all 88 randomized patients.

### 3.2 Efficacy

The 18-mo median OS rate (95% CI) was 63% (49–74%) in the ILIXA/SUN arm (*n* = 56) and 66% (46–80%) in the SUN arm (*n* = 30; *p* = 0.81). In high-risk patients, the 18-mo survival rates were 30% in the ILIXA/SUN arm and 38% in the SUN arm (*p* = 0.72). In intermediate-risk patients, the 18-mo survival rates were 77% in the ILIXA/SUN arm and 76% in the SUN arm (*p* = 0.97).

**Fig. 2:**
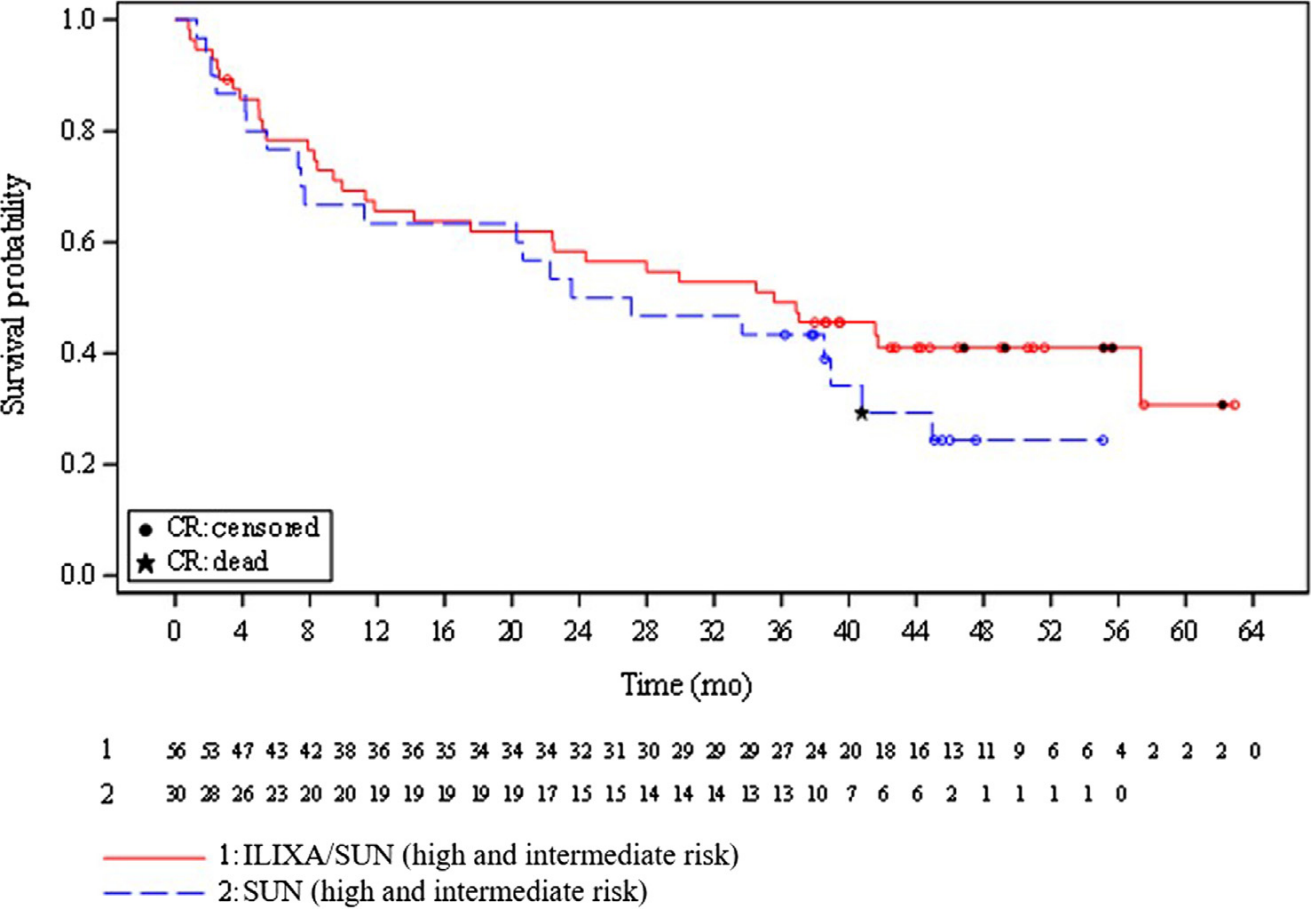
Kaplan-Meier estimate of survival for all patients. Survival probability (all patients) is displayed graphically using Kaplan-Meier, including summaries of the number of events (marked as a black star) and censored observations (marked as black circles). The red line represents ilixadencel and sunitinib strata (high and intermediate risk), and the blue line represents sunitinib strata (high and intermediate risk). The patients at risk for each stratum are indicated below the figure. CR = complete response.

After a minimum survival follow-up of 36.2 mo, the median OS (95% CI) was 35.6 (14.2–not available [NA]) mo in the ILIXA/SUN arm and 25.3 (7.7–40.8) mo in the SUN arm (HR 0.73, 95% CI 0.42–1.27; *p* = 0.25; Fig. 2). In the per-protocol population, the median OS (95% CI) was 41.8 (26.6–NA) mo in the ILIXA/SUN arm and 33.9 (11.3–45.2) mo in the SUN arm (HR 0.70, 95% CI 0.38–1.29; *p* = 0.24).

At the last survival follow-up, the number of patients still alive in the ILIXA/SUN arm was 22 of 55 (40.0%), compared with nine of 30 patients in the SUN arm (30.0%; odds ratio 2.0, 95% CI 0.8–5.1; *p* = 0.14). One additional patient was alive at 18 mo but was lost to subsequent survival follow-up.

The median OS (95% CI) for intermediate-risk patients was 41.7 (28.0–NA) mo in the ILIXA/SUN arm and 36.1 (7.7–44.9) mo in the SUN arm (HR 0.62, 95% CI 0.31–1.2; *p* = 0.16). In poor-risk patients, the median OS was reached at 10.6 (5.0–22.4) mo in the ILIXA/SUN arm and 9.3 (1.3–NA) mo in the SUN arm (HR 0.98, 95% CI 0.37–2.58; *p* = 0.96; Supplementary Fig. 1).

The median PFS from the start of sunitinib treatment in the ILIXA/SUN arm was 11.8 mo, as compared with 11.1 mo in the SUN arm (*p* = 0.55).

The confirmed ORR was 42.2% (19/45) in the ILIXA/SUN arm, including three patients with complete response (CR; Table 2). Among the three patients with a confirmed CR, the sum of tumor diameter in the target lesions were 22 mm (adrenal), 43 mm (lung × 2, muscle, and lymph node), and 22 mm (lung × 2), prior to treatment. Two additional patients in the ILIXA/SUN arm developed a CR at the last follow-up CT scan at 18 mo; one had stable disease until the CR and the other had a partial response after 3 mo until the CR was recorded at 18 mo. The ORR in the SUN arm was 24.0% (six/25; *p* = 0.13) with no confirmed CR. One patient in the SUN arm had a CR observed at the last 18-mo CT follow-up.

**Table 2:** Antitumor activity in IMDC intermediate- and high-risk patients after the start of sunitinib

	ILIXA/SUN group (*n* = 45^a^)	SUN group (*n* = 25)
Confirmed objective response rate (%)	19 (42.2)	6 (24.0)
Complete response	3 (6.7)	0 (0.0)
Partial response	16 (35.6)	6 (24.0)
Stable disease	11 (24.4)	10 (40.0)
Progressive disease	7 (15.6)	2 (8.0)
Unable to determine or not reported	9 (20.0)	7 (28.0)
Time to response (mo), median (95% CI)	2.9 (2.6–6.5)	4.2 (2.9–9.6)

CI = confidence interval; ILIXA = ilixadencel; IMDC = International Metastatic Renal Cell Carcinoma Database Consortium; SUN = sunitinib.

a One patient was not evaluable for response evaluation due to which no evaluation by imaging was performed.

The five ILIXA/SUN patients who achieved a CR (three confirmed and two nonconfirmed) were all alive at the latest survival follow-up, while the SUN patient with nonconfirmed CR died. In high-risk patients, the confirmed ORR was 38.5% for the ILIXA/SUN arm and 33.3% for the SUN arm (*p* = 0.83). In the intermediate-risk group, the corresponding rates were 43.8% in the ILIXA/SUN arm and 21.1% in the SUN arm, respectively (*p* = 0.1).

### 3.3 Sunitinib treatment: median duration, drug dose reduction, or interruption

The median duration of sunitinib treatment in the ILIXA/SUN arm was 6.9 (95% CI 6.0–9.3) mo, and it was 7.8 (95% CI 5.8–10.4) mo in the SUN arm. Dose reductions for sunitinib due to toxicity occurred in 17.4% (eight/46) of the ILIXA/SUN patients versus 24% (six/25) of the SUN patients, as shown in Supplementary Table 1. Of the ILIXA/SUN patients, 17.4% (eight/46) interrupted sunitinib temporarily and 19.6% (nine/46) discontinued it definitively due to toxicity, compared with 16% (four/25) and 20% (five/25) in the SUN arm, respectively (Supplementary Table 1).

### 3.4 Adverse events

AEs were reported in 93.1% (54/58) of ILIXA/SUN patients and in 90% (27/30) in the SUN arm. In total, 692 unique AEs were reported, that is, AE of a certain type was counted only once in each patient. In both groups, the most common AEs of any cause and the most common AEs related to treatment were fatigue, diarrhea, and nausea (see Table 3). Grade ≥3 anemia was slightly more common in patients in the ILIXA/SUN arm (5.2%) than in those in the SUN arm (3.3%). Anemia was not related to any vaccination procedures, but was judged due to disease progression (*n* = 2) and sunitinib treatment (*n* = 1) in the ILIXA/SUN arm and postoperative anemia in one patient in the SUN arm.

**Table 3:** Adverse events

Any AE	ILIXA/SUN (*N* = 58)		SUN (*N* =30)	
Preferred term^a^	Patients with occurrence, *n* (%)			
	Any grade	Grade 3 or 4	Any grade	Grade 3 or 4
Fatigue	14 (24.1)	1 (1.7)	8 (26.7)	0 (0)
Diarrhea	14 (24.1)	0 (0)	7 (23.3)	2 (6.7)
Nausea	14 (24.1)	1 (1.7)	7 (23.3)	0 (0)
Anemia	14 (24.1)	3 (5.2)	4 (13.3)	1 (3.3)
Hypertension	12 (20.7)	4 (6.9)	6 (20.0)	0 (0)
Asthenia	11 (19.0)	2 (3.5)	5 (16.7)	2 (6.7)
Pyrexia	11 (19.0)	2 (3.5)	4 (13.3)	0 (0)
Decreased appetite	10 (17.2)	0 (0)	5 (16.7)	0 (0)
Vomiting	14 (24.1)	2 (3.5)	0 (0)	0 (0)
Dysgeusia	9 (15.5)	0 (0)	5 (16.7)	0 (0)
Back pain	10 (17.2)	1 (1.7)	3 (10.0)	0 (0)
Stomatitis	6 (10.3)	1 (1.7)	6 (20.0)	0 (0)
Hypothyroidism	8 (13.8)	0 (0)	3 (10.0)	0 (0)
Palmar-plantar erythrodysesthesia syndrome	7 (12.1)	0 (0)	3 (10.0)	0 (0)
Blood creatinine increased	6 (10.3)	1 (1.7)	4 (13.3)	1 (3.3)
Constipation	7 (12.1)	0 (0)	2 (6.7)	0 (0)
Headache	7 (12.1)	0 (0)	1 (3.3)	0 (0)
Urinary tract infection	6 (10.3)	1 (1.7)	2 (6.7)	0 (0)
Arthralgia	5 (8.6)	1 (1.7)	3 (10.0)	0 (0)
Mucosal inflammation	6 (10.3)	0 (0)	1 (3.3)	0 (0)
Pain in extremity	5 (8.6)	0 (0)	2 (6.7)	0 (0)
Epistaxis	5 (8.6)	0 (0)	2 (6.7)	1 (3.3)
Abdominal pain upper	4 (6.9)	1 (1.7)	3 (10.0)	0 (0)
Dry skin	4 (6.9)	0 (0)	2 (6.7)	0 (0)
Hypercalcemia	4 (6.9)	1 (1.7)	2 (6.7)	2 (6.7)
Dyspnea	4 (6.9)	0 (0)	2 (6.7)	0 (0)
Oral pain	3 (5.2)	0 (0)	3 (10.0)	0 (0)
Dizziness	3 (5.2)	0 (0)	3 (10.0)	0 (0)
Procedural pain	3 (5.2)	0 (0)	3 (10.0)	1 (3.3)
Thrombocytopenia	5 (8.6)	1 (1.7)	0 (0)	0 (0)
Anxiety	5 (8.6)	0 (0)	0 (0)	0 (0)
Neutropenia	4 (6.9)	0 (0)	1 (3.3)	0 (0)
Cough	4 (6.9)	0 (0)	1 (3.3)	0 (0)
Hypotension	4 (6.9)	0 (0)	1 (3.3)	0 (0)
Abdominal pain	3 (5.2)	0 (0)	2 (6.7)	0 (0)
Platelet count decreased	2 (3.4)	1 (1.7)	3 (10.0)	1 (3.3)

AE = adverse event; ILIXA = ilixadencel; SUN = sunitinib.

a Preferred term used. Adverse events that occurred of any cause and occurred in ≥5% of patients in the treated population as per safety analysis. *N* = total number of patients in the group, used as the denominator for calculating percentage.

Grade 3 AEs occurred in 48 of all 88 patients enrolled (54.5%), 31 in the ILIXA/SUN arm and 17 in the SUN arm. Nine patients (10.2%) experienced a grade 4 AE, six in the ILIXA/SUN arm and three in the SUN arm. Overall, two patients (2.3%) discontinued the study due to an AE of any cause, one patient in the ILIXA/SUN arm and one in the SUN arm. Twelve patients (13.6%) discontinued due to death during the study, seven patients in the ILIXA/SUN arm and five in the SUN arm.

Treatment-related AEs of any grade occurred in 62 of 88 patients (70.5%). Sunitinib-related events of any grade occurred in 36 of 58 patients (62.1%) in the ILIXA/SUN arm and in 20 of the 30 patients (66.7%) in the SUN arm. Ilixadencel-related AEs of any grade occurred in 13 of 58 patients (22.4%) in the ILIXA/SUN arm.

Sunitinib-related grade 3 events occurred in 18 of 88 patients (20.5%) and grade 4 events in three patients (3.4%; see Table 4). Two patients developed a grade 3 AE that was attributed to ilixadencel, whereas five patients had a grade 3 AE that was considered related to sunitinib. One patient in the SUN arm died due to septic shock of abdominal origin that was attributed by the investigator to the treatment of sunitinib (1.1%). There were no grade 4 and 5 events related to treatment with ilixadencel.

**Table 4:** Treatment-related adverse events of grade ≥3^a^

Grade	AE related to	ILIXA/SUN (*N* = 58)	SUN (*N* = 30)	Total (*N* = 88)
		*n* (%)	*n* (%)	*n* (%)
3	SUN	13 (22.4)	5 (16.7)	18 (20.5)
	ILIXA	2 (3.4)	NA	2 (2.3)
4	SUN	1 (1.7)	2 (6.7)	3 (3.4)
	ILIXA	0 (0.0)	NA	0 (0.0)
5	SUN	0 (0.0)	1 (3.3)	1 (1.1)
	ILIXA	0 (0.0)	NA	0 (0.0)

AE = adverse event; ILIXA = ilixadencel; NA = not available; SUN = sunitinib.

a Common Terminology Criteria for Adverse Events version 5.0.

## 4 Discussion

This trial was a randomized, phase 2, open-label study comparing intratumoral ilixadencel followed by sunitinib with sunitinib monotherapy (standard of care at the time of the study), in patients with synchronous mRCC in the first-line setting. Upfront nephrectomy was performed in both groups (following ilixadencel vaccination in the experimental arm). The drug combination did not improve OS compared with sunitinib alone. These statistically negative OS data are in line with the data from a recently published phase 3 study in a patient population with synchronous mRCC using autologous monocyte-derived DCs loaded with mRNA from the autologous tumor plus sunitinib versus sunitinib alone, where no improvement in median OS was observed [17].

Nevertheless, ilixadencel in combination with sunitinib was associated with a numerically higher rate of confirmed responses (42.2%) including 6.7% CR (all alive at the last follow-up) compared with 24% with a confirmed response (none with a confirmed CR) in patients treated with sunitinib monotherapy. The observed responses seemed to be driven mainly by intermediate-risk patients. The short survival of poor-risk patients in both arms is consistent with more recent finding from phase 3 randomized clinical trials, indicating that upfront nephrectomy should be avoided in this subgroup due to the risk of rapidly progressive disease [18], [19]. Of possible interest, while the OS rate at 18 mo was comparable in both arms, the OS curves separated thereafter. This might indicate a possibility of late responses with prolonged duration in the ILIXA/SUN arm. Indeed, remarkably late CRs (18 mo) were noted in two patients treated with the combination. Furthermore, the nearly two-fold higher rate of confirmed objective responses in the ILIXA/SUN patients, as compared with sunitinib alone, may be clinically meaningful. Our hypothesis is that ilixadencel induces a systemic and sustained activation of tumor-specific T cells and that the subsequent blockade of different VEGF-dependent immunosuppressive mechanisms by sunitinib will unleash the full antitumor potential of an ilixadencel-induced systemic and sustained tumor-specific, T-cell response. Such a proposed synergistic mechanism is supported by data from others showing that sunitinib works in concert with immune primers and anticancer vaccines, including DC-based vaccines in preclinical tumor models [20], [21], [22].

The addition of ilixadencel to sunitinib did not increase toxicity, and the safety profile was consistent with previous experience with ilixadencel in combination with TKIs in mRCC [15], HCC [14], and gastrointestinal stromal tumors [23]. The schedule of sunitinib was in line with previous randomized trials with sunitinib [5], [9]. The incidence of grade 3 and 4 events was similar in both arms, whereas no treatment-related deaths occurred in the ILIXA/SUN arm.

The present study has several limitations, including a small sample size and absence of blinding, as expected for a phase 2 trial using an interventional procedure. A non-negligible proportion did not proceed to sunitinib treatment, introducing a potential selection bias. Moreover, exploratory in its design, the trial was not adequately powered to detect modest differences in survival. The present study population of patients with synchronous metastases could possibly have diminished sensitivity, in particular since both arms included a substantial number of high-risk patients who may not have benefitted from the treatment approach with upfront nephrectomy [18], [19]. Moreover, since patient survival was the only postprogression parameter recorded, potential differences in second-line treatment between the groups cannot be excluded. In addition, the standard of care has evolved since the time this trial was conceived, and sunitinib monotherapy is currently recommended only as first-line medical therapy in intermediate- and poor-risk mRCC patients who cannot receive or tolerate immune checkpoint inhibition [7]. The present study was limited to synchronous mRCC due to the study design, indicating that most patients would have remained unbenefited even with positive study results. Nevertheless, patients with synchronous metastatic RCC represent a vulnerable group of RCC patients in whom good treatment options were largely lacking, motivating our trial design. Future trials of ilixadencel in mRCC should therefore use ICI-based combinations as backbone regimens. Based on the results of the present study, The US Food and Drug Administration has recently supported further development of the ilixadencel and sunitinib combination with a Regenerative Medicine Advanced Therapy designation [24].

## 5 Conclusions

In conclusion, despite that this trial failed to meet its primary endpoints, it showed the combination of ilixadencel with sunitinib to be safe and associated with promising overall and CR rates, compared with sunitinib alone. Future investigations of ilixadencel with regimens combining ICIs and TKIs in the study population are warranted.

  ***Author contributions*:** Magnus Lindskog had full access to all the data in the study and takes responsibility for the integrity of the data and the accuracy of the data analysis.

*Study concept and design*: Lindskog, Suenaert, Karlsson-Parra, Ljungberg.

*Acquisition of data*: Lindskog, Laurell, Kjellman, Melichar, Rey, Zieliński, Villacampa, Bigot, Zoltan, Parikh, Alba, Jellvert, Flaskó, Gallardo, Caparrós, Purkalne, Suenaert, Karlsson-Parra, Ljungberg.

*Analysis and interpretation of data*: Lindskog, Suenaert, Karlsson-Parra, Ljungberg.

*Drafting of the manuscript*: Lindskog, Suenaert.

*Critical revision of the manuscript for important intellectual content*: Lindskog, Suenaert, Karlsson-Parra, Ljungberg.

*Statistical analysis*: Lindskog, Suenaert, Karlsson-Parra, Ljungberg.

*Obtaining funding*: None.

*Administrative, technical, or material support*: Lindskog, Suenaert, Karlsson-Parra.

*Supervision*: Lindskog, Suenaert, Karlsson-Parra, Ljungberg.

*Other*: None.

  ***Financial disclosures:*** Magnus Lindskog certifies that all conflicts of interest, including specific financial interests and relationships and affiliations relevant to the subject matter or materials discussed in the manuscript (eg, employment/affiliation, grants or funding, consultancies, honoraria, stock ownership or options, expert testimony, royalties, or patents filed, received, or pending), are the following: Alex Karlsson-Parra and Peter Suenaert report ownership of stocks in Immunicum AB. Alex Karlsson-Parra is an Immunicum employee, and Peter Suenaert is an Immunicum consultant. All other authors report no conflict of interest.

  ***Funding/Support and role of the sponsor*:** The research reported is sponsored by Immunicum AB.

  ***Ethics statement*:** This study was approved by the Independent Ethics Committee and the Institutional Review Board prior to inclusion of patients. Patients provided written informed and signed consent before participation.

  ***Data sharing*:** No identifiable data will be shared and should not be requested. The datasets used and/or analyzed during the current study are available from the corresponding author on reasonable request.

  ***Acknowledgments:*** We thank the patients who participated in this study and their families, the clinicians, and staff at each center who looked after these patients. We also thank the teams at the contract research organizations TFS Trial Form Support and Accelovance, as well as the team at SMS Oncology for providing support throughout the study. The writing assistance of Emilia Heimann at Immunicum AB is gratefully acknowledged.
