# Supplementary material for: Ilixadencel, a Cell-based Immune Primer, plus Sunitinib Versus Sunitinib Alone in Metastatic Renal Cell Carcinoma: A Randomized Phase 2 Study
Source: Eur Urol Open Sci. 2022 Apr 26;40:38–45. doi: 10.1016/j.euros.2022.03.012 (PMC9142735; doi:10.1016/j.euros.2022.03.012)
Supplement: Supplementary data 1 [file mmc1.docx]

## SUPPLEMENTARY MATERIAL

**
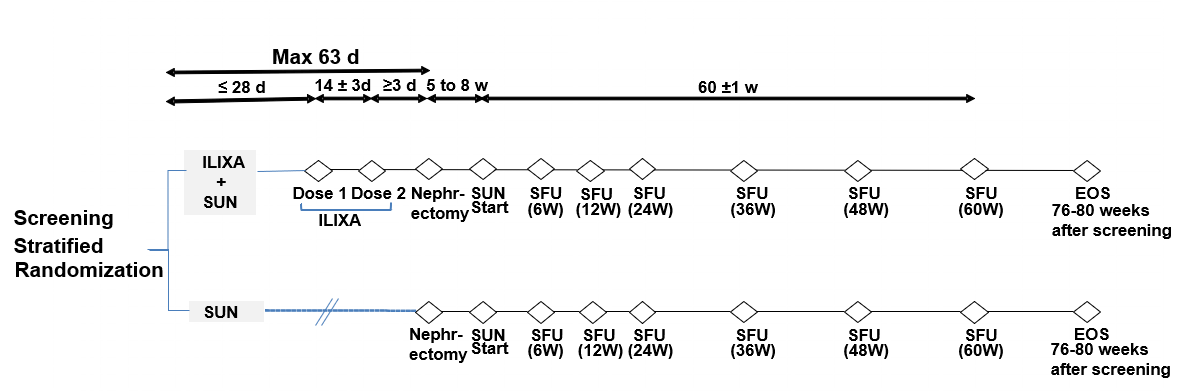
**

**Supplementary Figure 1. Assessment of tumor response at screening, baseline and each follow-up visit.**

*CT scans for central assessment of tumor response were performed at screening, sunitinib start i.e., 5 to 8 weeks after nephrectomy (baseline), at each sunitinib follow-up (SFU) visit (6, 12, 24, 36, 48 and 60 weeks) and at End-of-Study visit (78 weeks after screening).*

**
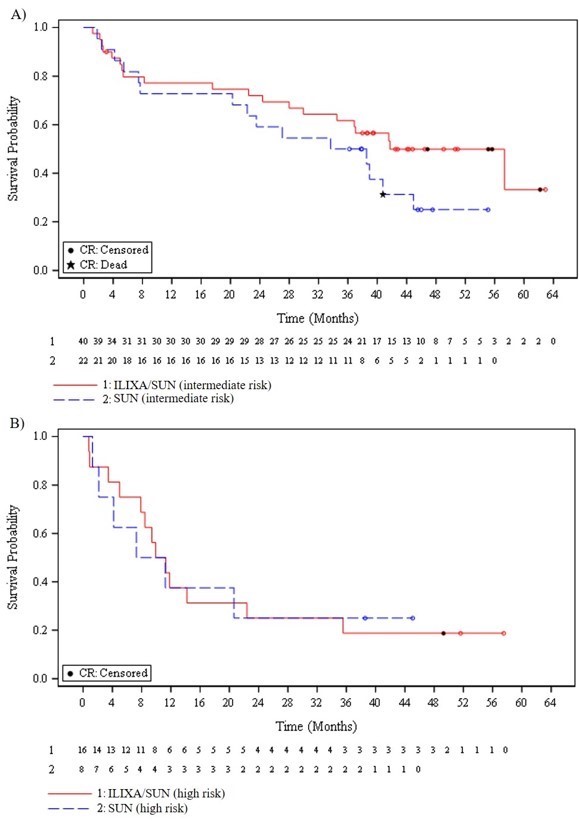
**

**Supplementary Figure 2. Kaplan-Meier estimate of survival for patients with intermediate and poor prognosis.**

*Survival probability for A) intermediate and B) high risk groups are displayed graphically using Kaplan-Meier, including summaries of number of events (marked as a black star) and censored observations (marked as a black circle). The red line represents ilixadencel and sunitinib strata and the blue line represents sunitinib strata. The patients at risk are indicated below figure for each stratum.*

**Supplementary Table 1. Sunitinib dose reduced, interrupted or stopped in both treatment groups due to adverse events*.***

| **Sunitinib:** | **IMDC risk group** | **ILIXA/SUN (N=46)** | **SUN (N=25)** |
| --- | --- | --- | --- |
|  |  | **n (%)** | **n (%)** |
| **Dose reduced** | Intermediate | 5 (10.9) | 5 (20) |
|  | High | 3 (6.5) | 1 (4) |
|  | **Total** | 8 (17.4) | 6 (24) |
|  | | | |
| **Treatment interrupted** | Intermediate | 2 (4.3) | 3 (12) |
|  | High | 6 (13) | 1 (4) |
|  | **Total** | 8 (17.4) | 4 (16) |
|  | | | |
| **Treatment stopped** | Intermediate | 5 (10.9) | 3 (12) |
|  | High | 4 (8.7) | 2 (8) |
|  | **Total** | 9 (19.6) | 5 (20) |
